# Early Effectiveness of Four SARS-CoV-2 Vaccines in Preventing COVID-19 among Adults Aged ≥60 Years in Vojvodina, Serbia

**DOI:** 10.3390/vaccines10030389

**Published:** 2022-03-03

**Authors:** Vladimir Petrović, Vladimir Vuković, Miloš Marković, Mioljub Ristić

**Affiliations:** 1 Department of Epidemiology, Faculty of Medicine, University of Novi Sad, 21000 Novi Sad, Serbia; vladimir.petrovic@izjzv.org.rs (V.P.); mioljub.ristic@mf.uns.ac.rs (M.R.); 2 Institute of Public Health of Vojvodina, 21000 Novi Sad, Serbia; 3 Department of Immunology, Faculty of Medicine, Institute of Microbiology and Immunology, University of Belgrade, 11000 Belgrade, Serbia; milos.markovic@med.bg.ac.rs

**Keywords:** COVID-19, vaccination, effectiveness, elderly, public health policy

## Abstract

Real-world evidence of the vaccine effectiveness (VE) of different COVID-19 vaccines is needed in order to better shape vaccine recommendations and policies and increase vaccine acceptance, especially among vulnerable populations such as the elderly. We analyzed the early effectiveness of four COVID-19 vaccines, namely BNT162b2, BBIBP-CorV, Gam-COVID-Vac and ChAdOx1 nCoV-19 in population aged ≥60 years for symptomatic, mild and severe COVID-19, in the period January–April 2021 in Vojvodina, a northern province of Serbia. Incidence rates of SARS-CoV-2 infection were calculated using data from the provincial COVID-19 surveillance registry, and vaccination coverage data were obtained from the nationwide registry of administered COVID-19 vaccines. During the observation period, 134,535 subjects aged ≥60 years were fully vaccinated, of whom 87.7% received BBIBP-CorV, 7.1% BNT162b2 and 5.2% Gam-COVID-Vac vaccines. The estimated VE in fully vaccinated persons was 86.9% (95% CI, 86–87.7) for BBIBP-CorV, 95% (95% CI, 92.4–96.7) for Gam-COVID-Vac and 99% (95% CI, 97.8–99.5) for BNT162b2, while VE after the first dose of ChAdOx1 nCoV-19 was 88.6% (95% CI, 80.5–93.4). Estimates were similar when stratifying the analyses to severe and mild SARS-CoV-2 infections. Our analysis provides evidence of high early effectiveness of BNT162b2, BBIBP-CorV, Gam-COVID-Vac and ChAdOx1 nCoV-19 in elderly people in preventing symptomatic, severe and mild COVID-19 disease, particularly after being fully vaccinated.

## 1. Introduction

The ongoing coronavirus disease 2019 (COVID-19) pandemic, caused by severe acute respiratory syndrome coronavirus 2 (SARS-CoV-2), has major consequences for the health of the population as well as a huge impact on healthcare systems globally [1]. The development of an effective vaccine has been a global priority and different vaccine platforms have been delivered [2]. Worldwide, vaccination campaigns with newly approved vaccines were rapidly implemented to induce protective immunity in vulnerable populations (especially among those aged ≥60 years) and to contain the spread of SARS-CoV-2. In Serbia, the COVID-19 vaccination campaign started on 24 December 2020, after the Serbian Medicines Agency approved the Pfizer-BioNTech BNT162b2 (Comirnaty^®^) vaccine. In the following weeks, three more vaccines, namely Sinopharm BBIBP-CorV (Vero Cell^®^), Gamaleya Research Institute Gam-COVID-Vac (Sputnik V^®^) and Oxford-AstraZeneca ChAdOx1 nCoV-19 AZD1222 (Vaxzevria^®^) were approved by the same agency [3]. These vaccines have different mechanisms of action and differences in terms of logistics, which are described elsewhere, but they all demonstrated satisfactory levels of safety and efficacy in controlled trial settings [4,5,6,7]. The vaccination campaign in Serbia initially targeted the elderly population, together with healthcare workers, and residents in long-term care facilities [8]. Following the BNT162b2 vaccine, BBIBP-CorV, Gam-COVID-Vac and ChAdOx1 nCoV-19 gradually became available and were offered on a voluntary basis to all adult individuals in Serbia. The recommended administration was two doses 21 days apart for the BNT162b2, BBIBP-CorV and Gam-COVID-Vac vaccines, and 12 weeks apart for the ChAdOx1 nCoV-19 vaccine.

Apart from evidence from clinical trials, real-world surveillance data on COVID-19 vaccine effectiveness (VE) are needed in order to better shape vaccine recommendations and policies, overcome vaccination hesitancy and increase vaccine acceptance, especially among populations such as the elderly that are particularly vulnerable to severe SARS-CoV-2 infections, complications and worse prognoses [9,10]. At the same time, this population might be under-represented in clinical trials [11,12]. Evidence from population-level data in regard to the effectiveness of different COVID-19 vaccines in the elderly is still insufficient, and much remains to be understood after widespread vaccination. Moreover, studies particularly aimed at estimating the effectiveness of BBIBP-CorV and Gam-COVID-Vac vaccines are still largely lacking.

We have analyzed the early effectiveness of all four COVID-19 vaccines available in Serbia in the period from January to April 2021, after application of the first and the second vaccine dose, for symptomatic COVID-19, as well as mild and severe forms of the disease, in population aged ≥60 years. This allowed us to present effectiveness in comparative terms using different vaccine platforms in the same population.

## 2. Materials and Methods

We used data from the Autonomous Province of Vojvodina (Vojvodina in subsequent text) COVID-19 surveillance registry which contains socio-demographic and epidemiological characteristics of all COVID-19 laboratory-confirmed cases, obtained by RT-PCR or antigen rapid diagnostic test (Ag-RDT) [13], from the beginning of the epidemic in March 2020. We only considered subjects aged ≥60 years at the time of the COVID-19 RT-PCR or Ag-RDT positive test, in the period between 24 December 2020 and 28 April 2021. This registry also includes information on the clinical presentation of COVID-19 at the date of attending medical care, the patient’s hospitalization status as well as disease outcome (active, recovery or death). Clinical presentation of COVID-19 was defined as symptomatic if patients, during the time of positivity, experienced any of the general infective, respiratory or digestive symptoms related to COVID-19. It was classified as mild when patients experienced symptoms without clinical or radiological signs of pneumonia, and as severe when pneumonia was confirmed and oxygenation was needed.

To obtain the vaccination status of COVID-19 cases (type and dose(s) of vaccine and date(s) of their administration), data from the surveillance registry were linked to the nationwide registry of administered COVID-19 vaccines using the Unique Master Citizen Number, a personal digit code which is assigned to all citizens of Serbia. All vaccinated individuals received the same type of vaccine for their first and second doses and followed the recommended time schedule between doses, since only the homologous regimen of COVID-19 vaccination was applied in the early phase of the immunization campaign in Serbia.

All subjects who received the first dose between 24 December 2020 and 24 February 2021 were considered in the analyses. For evaluation of the effectiveness of the first dose of the above-mentioned vaccines, the follow-up started 14 days after receiving the first dose in order to take into account the time needed for seroconversion, and lasted up until 14 days after application of the second dose, i.e., 35 days after receiving the first dose. For evaluation of the effectiveness of the second dose, the follow-up started 14 days from application of the second dose and lasted four weeks. Of note, considering the longer period (12 weeks) between the two doses for the ChAdOx1 nCoV-19 vaccine and the late arrival of this vaccine to Serbia (the end of February 2021), as well as the follow-up period of four months (from January to April 2021) in this study, vaccine effectiveness for the ChAdOx1 nCoV-19 vaccine was estimated only for the first dose.

For patients with confirmed COVID-19, based on the number of vaccine doses received before the date of symptom onset or that of a positive COVID-19 test (whichever occurred earlier), they were classified as follows: unvaccinated, if no vaccine dose was received; partially vaccinated, if they received one dose; and fully vaccinated, if both vaccine doses were administered ≥14 days before the reference date. Considering the time required for seroconversion after vaccination, people with COVID-19 cases who received the first dose of a vaccine <14 days before the reference date were considered unvaccinated. Likewise, those who were positive before 14 days elapsed after the second dose were considered partially vaccinated. Subjects with a positive test at any time before 7 January 2021 (the maximum incubation period of 14 days after the beginning of the follow-up period, i.e., 24 December 2020) were excluded from the analyses. Finally, the population of susceptible unvaccinated people was defined as the difference between the estimated population aged ≥60 years in Vojvodina and the vaccinated persons in this population.

### 2.1. Data Analysis

Risk ratio (RR) with corresponding 95% confidence interval (95% CI) was calculated comparing the risk in those vaccinated (the share of vaccinated people that contracted the virus) with the risk in unvaccinated (the share of unvaccinated positive people of all unvaccinated). VE and 95% CI for the first and second doses were computed as a difference 1-RR, and were expressed as a percentage. VE estimates were stratified by different SARS-CoV-2 vaccines and by the severity of COVID-19 clinical presentation.

### 2.2. Ethical Statement

No ethical approval was required since the study consisted of the analysis of secondary data collected as part of a routine COVID-19 surveillance of SARS-CoV-2-positive cases and vaccine administration.

## 3. Results

During the first study period (within the follow-up period from 24 December 2020 to 31 March 2021), 139,858 persons aged ≥60 years were analyzed after receiving one dose of a vaccine, while during the observation period for the second dose (28 January 2021 up to 28 April 2021), a total of 134,535 subjects from the same age category were considered as fully vaccinated, and were included in the analyses of VE. Among subjects vaccinated with the first dose, 86% received BBIBP-CorV, 7% BNT162b2, 5.1% Gam-COVID-Vac and 1.9% ChAdOx1 nCoV-19 vaccines. As for those who received the second dose, the percentages were similar (87.7% were vaccinated with BBIBP-CorV, 7.1% with BNT162b2 vaccine and 5.2% with Gam-COVID-Vac, while none of the subjects received the second dose of ChAdOx1 nCoV-19 vaccine due to a longer dosing interval of 12 weeks). During the observation period, after the first dose in all vaccinated persons, there were 840 patients with laboratory-confirmed COVID-19 (0.6%), among whom 725 (0.5%) patients had mild and 115 (0.1%) had severe form of disease. In addition, there were 830 (0.6%) and 162 (0.1%) vaccinated patients with mild and severe COVID-19, respectively, who were infected in the period of 14 days from the second dose (Table S1).

A detailed description of VE with corresponding 95% confidence interval after the first dose across different vaccine types is presented in Table 1.

The incidence of COVID-19 in vaccinated people was 6.0 per 1000 people and ranged from 2.1 in those vaccinated with BNT162b2 to 6.5 per 1000 people in those vaccinated with BBIBP-CorV. For the follow-up period for evaluation of the effectiveness of the first dose, incidence of COVID-19 cases in the non-vaccinated elderly population was 44.1 per 1000 people. Calculated RR was 0.14, ranging from 0.05 in those vaccinated with BNT162b2 to 0.15 in those vaccinated with BBIBP-CorV. For partially vaccinated elderly people (14 to 35 days after receiving the first dose), the VE in preventing COVID-19 was estimated at 86.4% in all those vaccinated, and varied by vaccine type, from 85.4% in those vaccinated with BBIBP-CorV, 88.6% with ChAdOx1 nCoV-19, 90.9% with Gam-COVID-Vac and up to 95.2% in elderly people who were partially vaccinated with BNT162b2. As for VE in regard to the clinical presentation of disease, the VE of the first dose in preventing a mild form of COVID-19 was 84.7%, being highest for the BNT162b2 vaccine (95.5%); while in preventing severe COVID-19, it was 92%, and ranged from 91.7% for BBIBP-CorV to 94% for BNT162b2.

Considering the follow-up period for evaluation of the effectiveness of the second dose, incidence of COVID-19 in two-dose vaccinees was 7.2 per 1000 people, while the incidence in the non-vaccinated elderly population was 62.3 per 1000 people (Table 2).

Effectiveness of vaccination with the two-dose regimen in preventing COVID-19 was 88.4% in total, but varied between vaccines, from a demonstrated 86.9% for those fully vaccinated with BBIBP-CorV, through 95% for vaccinated subjects with Gam-COVID-Vac, up to 99% for those who received two doses of BNT162b2. Estimates were similar when stratifying the analyses to mild and severe SARS-CoV-2 infections. For instance, VE after the second dose in preventing the most severe cases was estimated at 90.5% for BBIBP-CorV, 95.9% for Gam-COVID-Vac and 97% for BNT162b2. As could be expected, VE was somewhat lower in regard to protection against mild COVID-19, for which the overall effectiveness of the second dose of all three vaccines together was 87.5%.

## 4. Discussion

This is the first study that evaluates the VE of four different vaccines against COVID-19 among participants aged ≥60 years in our country. It is also one of the rare studies that assessed the population-based effectiveness of the BBIBP-CorV and Gam-COVID-Vac vaccines. Our analysis suggests that all four vaccines had high effectiveness in preventing SARS-CoV-2 infection in the elderly for the short time intervals following their administration. During the period from 14 to 35 days after the first dose, the estimated VE for symptomatic infection was 85.4% for BBIBP-CorV, 88.6% for ChAdOx1 nCoV-19, 90.9% for Gam-COVID-Vac and 95.2% for BNT162b2. The estimated VE in fully vaccinated persons during the first month was expectedly higher, i.e., 86.9%, 95% and 99%, for BBIBP-CorV, Gam-COVID-Vac and BNT162b2, respectively (no subjects in our study were vaccinated with two doses of ChAdOx1 nCoV-19). However, the high VE of the first dose of all four analyzed vaccines that was similar to the VE of the second dose is somewhat surprising, and could be explained by the short follow-up period as well as by the change in the predominant SARS-CoV-2 variant in Vojvodina during the study period. Namely, during follow-up for the first dose (January and February 2021), the Wuhan variant was still widely present, while throughout the second study period (March and April 2021) the Alpha variant (also known as lineage B.1.1.7) became predominant.

The main objective of vaccination in elderly persons is to protect them against severe COVID-19 and a lethal outcome. Despite a weaker response, and a reduction in VE in the elderly due to immunosenescence, general frailty, possible comorbidities and malnutrition [14], our results are mostly in line with previous findings with satisfactory COVID-19 VE in this population. A recent Spanish registry-based cohort study of long-term care-facility residents reported the VE of BNT162b2 in the elderly to be 81.8% (95% CI, 81–82.7) [15]. Another study from England, using observational data and conducted in a period that overlaps with our study period (from 8 December 2020 to 19 February 2021), found VE of a single dose of BNT162b2 in preventing symptomatic disease 28 days after vaccination to be about 70% (95% CI, 59–78) in those aged ≥70 years, while in persons >80 years of age, the estimated VE was 89% 14 days after the second dose [16]. In the same study, the effectiveness of a single-dose ChAdOx1 nCoV-19 vaccine was 60% (95% CI, 41–73) among those aged ≥70 years. On the other hand, a cohort study from the United Kingdom reported an effectiveness of 68% after a single dose of Oxford-AstraZeneca vaccine (at 35 to 48 days post-vaccination) in residents (≥65 years) of long-term care facilities [17].

In contrast to BNT162b2 and ChAdOx1 nCoV-19 vaccines, only a few studies evaluated the effectiveness of Gam-COVID-Vac [18,19,20] and BBIBP-CorV vaccines [12,20] in elderly populations. A randomized, controlled phase 3 trial conducted in Russia showed an efficacy of 87.6% (95% CI, 81.1–91.8) 14 days after vaccination with one dose of Gam-COVID-Vac, and an efficacy of 91.1% (95% CI, 83.8–95.1) for prevention of symptomatic COVID-19 after two doses, while in participants aged ˃60 years, from 21 days after the first dose (the day of administration of dose 2), vaccine efficacy reached 91.8% (95% CI, 67.1–98.3) [18]. A recently published retrospective cohort study from Argentina evaluated VE of the first component of Gam-COVID-Vac in elderly people aged 60–79 and reported an effectiveness of 78.6% (95% CI, 74.8–81.7) in preventing symptomatic COVID-19 [19]. In another recently published study from Hungary, the adjusted effectiveness of Gam-COVID-Vac for prevention of SARS-CoV-2 infection in persons older than 55 years ranged between 84.8% and 90.9% depending on age, while its effectiveness against COVID-19-related death was higher than 95% in all analyzed age groups (from 95.4% in the 74–85 years group to 100% in those 85-years-old and over) [20]. As for inactivated BBIBP-CorV vaccine, an interim analysis of a randomized phase 3 trial of BBIBP-CorV conducted in the United Arab Emirates and Bahrain among adults aged 18 years reported an efficacy of 72.8% (95% CI, 58.1–82.4) against symptomatic COVID-19 and 100% against severe disease 14 days after a second dose of BBIBP-CorV [12], although the number of subjects older than 60 years was very low in both cohorts. In the previously mentioned study from Hungary, the adjusted effectiveness of BBIBP-CorV for prevention of SARS-CoV-2 infection ranged between 43.1% in persons older than 85 years and 71.1% in the group of 65–74 years old, while the effectiveness against COVID-19-related death ranged between 67.3% in those 85+ and 91.1% in the 65–74 years group [20]. On the other hand, estimated VE for other inactivated SARS-CoV-2 vaccines (CoronaVac, Sinovac) among subjects aged ≥60 years in Chile was 66.6% (95% CI, 65.4 to 67.8) for the prevention COVID-19 and 85.3% (95% CI, 84.3 to 86.3) for the prevention of hospitalization (severe COVID-19) [21]. Finally, a recently published study from our research group investigating immunogenicity of BNT162b2, BBIBP-CorV and Gam-COVID-Vac vaccines on a small sample from the same population as in this study, demonstrated a high proportion of seropositive participants in the age group ≥60 years on the 28th day after the second dose of BNT162b2 or Gam-COVID-Vac (100% each), while for BBIBP-CorV it was somewhat lower (85.7% seroconverted in age group 60–69 years, 90.9% in 70–79, while there was one single seronegative participant in the 80+ age category) [22]. Moreover, the antibody levels detected in their sera were in line with the estimated effectiveness of these three vaccines in the current study, with the highest values observed in older persons vaccinated with BNT162b2, and the lowest antibody levels measured in those vaccinated with BBIBP-CorV.

In general, estimates of the VE from the available literature are lower than in our study. Even though comparisons of findings across different studies are challenging, the observed discrepancy could be ascribed to different periods of evaluation, differences in circulating virus variants, levels of SARS-CoV-2 transmission (prevalence of cases) in the community, characteristics of the included population, testing protocols, case definition and surveillance procedures, and heterogeneity of study methodology (e.g., the timing of the post-vaccination follow-up was also variable) [23]. Because of the short follow-up period after vaccination with one and/or two doses of observed vaccines, it was too early to assess the VE for mortality so these findings are missing in the present study. At the same time, our study has some other limitations related to the observational nature and design of the study which might affect the estimates. For instance, the availability and practice of COVID-19 testing, evaluation of the severity of symptoms and course of disease can affect the prevalence of cases and influence the VE estimates reported here. Additionally, our estimates are based on the administrative data collected during routine surveillance of COVID-19 cases, which might be affected by some biases, in particular when reporting the symptoms and severity of the disease. Still, at the time, these administrative data represented the best available source of population-based data on this topic in our country. Next, the elderly tend to follow medical advice and have a higher adherence to preventive measures such as wearing a mask, social distancing, hand hygiene, etc. Finally, due to a difference in the prevalence of cases and circulating virus variants across time periods and territories, our findings may not be suitable for extrapolation to other regions and countries. In line with this, the major limitation of our study is that our findings do not include VE against SARS-CoV-2 variants other than Alpha (e.g., Delta or Omicron variants) that were not circulating during the study period, but are known for being able to significantly evade neutralization and thus affecting the effectiveness of the vaccines.

## 5. Conclusions

Despite all above-mentioned limitations, this population-based evaluation of VE in a non-controlled setting provides evidence of high early effectiveness of four available vaccines (BNT162b2, BBIBP-CorV, Gam-COVID-Vac and ChAdOx1 nCoV-19) in elderly people in preventing severe and mild COVID-19 disease, particularly after being fully vaccinated. These findings are relevant for (re)evaluating the benefits of different vaccine platforms for elderly populations that may often respond inadequately to vaccination. Nevertheless, the vaccinated elderly should continue to follow transmission-preventing measures such as mask-wearing, disinfection and physical distancing until vaccine uptake in other age categories reaches high levels.

## Figures and Tables

**Table 1:** Estimated effectiveness of the first dose of SARS-CoV-2 vaccines against COVID-19 outcomes, by clinical presentation and type of vaccine.

Clinical Presentation	Vaccine	Incidence in Single-Dose Vaccinees (per 1000 People)	Incidence in Susceptible Population (per 1000 People)	RR	95% CI		Effectiveness of the First Dose (%)	95% CI (%)	
COVID-19 (overall)	BBIBP-CorV	6.5	44.1	0.15	0.14	0.16	85.40	84.30	86.40
	ChAdOx1 nCoV-19	5.0	44.1	0.11	0.07	0.20	88.60	80.50	93.40
	BNT162b2	2.1	44.1	0.05	0.03	0.07	95.20	92.60	96.80
	Gam-COVID-Vac	4.0	44.1	0.09	0.06	0.13	90.90	86.90	93.60
	All vaccines	6.0	44.1	0.14	0.13	0.15	86.40	85.40	87.30
Mild COVID-19	BBIBP-CorV	5.6	33.9	0.17	0.15	0.18	83.40	82.10	84.70
	ChAdOx1 nCoV-19	4.2	33.9	0.13	0.07	0.23	87.50	77.40	93.10
	BNT162b2	1.5	33.9	0.05	0.03	0.08	95.50	92.50	97.30
	Gam-COVID-Vac	3.3	33.9	0.10	0.07	0.15	90.20	85.30	93.40
	All vaccines	5.2	33.9	0.15	0.14	0.17	84.70	83.50	85.80
Severe COVID-19	BBIBP-CorV	0.8	10.2	0.08	0.07	0.10	91.70	89.90	93.20
	ChAdOx1 nCoV-19	0.8	10.2	0.08	0.02	0.30	92.50	69.80	98.10
	BNT162b2	0.6	10.2	0.06	0.03	0.13	94.00	86.70	97.30
	Gam-COVID-Vac	0.7	10.2	0.07	0.03	0.16	93.20	83.60	97.20
	All vaccines	0.8	10.2	0.08	0.07	0.10	92.00	90.30	93.30

RR = risk ratio; 95% CI = 95% confidence interval.

**Table 2:** Estimated effectiveness of the second dose of SARS-CoV-2 vaccines against COVID-19 outcomes, by clinical presentation and type of vaccine.

Clinical Presentation	Vaccine	Incidence in Two-Dose Vaccinees (per 1000 People)	Incidence in Susceptible Population (per 1000 People)	RR	95% CI		Effectiveness of the Second Dose (%)	95% CI (%)	
COVID-19 (overall)	BBIBP-CorV	8.2	62.3	0.13	0.12	0.14	86.90	86.00	87.70
	BNT162b2	0.6	62.3	0.01	0.01	0.02	99.00	97.80	99.50
	Gam-COVID-Vac	3.1	62.3	0.05	0.03	0.08	95.00	92.40	96.70
	All vaccines	7.2	62.3	0.12	0.11	0.12	88.40	87.60	89.10
Mild COVID-19	BBIBP-CorV	6.9	48.5	0.14	0.13	0.15	85.80	84.80	86.80
	BNT162b2	0.2	48.5	0.00	0.00	0.02	99.60	98.30	99.90
	Gam-COVID-Vac	2.6	48.5	0.05	0.03	0.08	94.70	91.60	96.70
	All vaccines	6.1	48.5	0.13	0.12	0.13	87.50	86.60	88.40
Severe COVID-19	BBIBP-CorV	1.3	13.8	0.10	0.08	0.11	90.50	88.90	91.90
	BNT162b2	0.4	13.8	0.03	0.01	0.08	97.00	91.90	98.90
	Gam-COVID-Vac	0.6	13.8	0.04	0.02	0.11	95.90	89.00	98.50
	All vaccines	1.2	13.8	0.09	0.07	0.10	91.40	90.00	92.70

RR = risk ratio; 95% CI = 95% confidence interval.

## Data Availability

The data that support the findings of this study are available from the corresponding author upon reasonable request.

## Supplementary Materials

The following supporting information can be downloaded at: https://www.mdpi.com/article/10.3390/vaccines10030389/s1, Table S1: Characteristics of the analyzed population, by clinical presentation of COVID-19 and the type of administered vaccine.
