# Supplementary material for: Early Effectiveness of Four SARS-CoV-2 Vaccines in Preventing COVID-19 among Adults Aged ≥60 Years in Vojvodina, Serbia
Source: Vaccines (Basel). 2022 Mar 3;10(3):389. doi: 10.3390/vaccines10030389 (PMC8954033; doi:10.3390/vaccines10030389)
Supplement: Supplementary file 1 [file vaccines-10-00389-s001.zip › vaccines-1576294-supplementary.pdf]

Table S1. Characteristics of the analyzed population, by clinical presentation of COVID-19 and the type of administered vaccine.

	Population of vaccinated with one dose, N (%)	Vaccinated population infected in the period from 14 days after the first dose until 14 days after the second dose, N (%)	Susceptible population of unvaccinated, N	Susceptible population of unvaccinated infected, N	Population of vaccinated with two doses, N (%)	Vaccinated population infected in the period after 14 days after the second dose, N (%)	Susceptible population of unvaccinated, N	Susceptible population of unvaccinated infected, N
**COVID-19 overall**								
BBIBP-CorV	120,236 (85.97)	777 (92.50)	276,590	12,204	117,914 (87.65)	964 (97.18)	241,166	15,021
ChAdOx1 nCoV-19	2594 (1.86)	13 (1.55)	276,590	12,204	0	N/A	241,166	15,021
BNT162b2	9836 (7.03)	21 (2.50)	276,590	12,204	9584 (7.12)	6 (0.60)	241,166	15,021
Gam-COVID-Vac	7192 (5.14)	29 (3.45)	276,590	12,204	7037 (5.23)	22 (2.22)	241,166	15,021
All vaccines	139,858 (100)	840 (100)	276,590	12,204	134,535 (100)	992 (100)	241,166	15,021
**Mild COVID-19**								
BBIBP-CorV	120,236 (85.97)	675 (93.10)	276,590	9378	117,914 (87.65)	810 (97.59)	241,166	11,694
ChAdOx1 nCoV-19	2594 (1.86)	11 (1.52)	276,590	9378	0	N/A	241,166	11,694
BNT162b2	9836 (7.03)	15 (2.07)	276,590	9378	9584 (7.12)	2 (0.24)	241,166	11,694
Gam-COVID-Vac	7192 (5.14)	24 (3.31)	276,590	9378	7037 (5.23)	18 (2.17)	241,166	11,694
All vaccines	139,858 (100)	725 (100)	276,590	9378	134,535 (100)	830 (100)	241,166	11,694
**Severe COVID-19**								
BBIBP-CorV	120,236 (85.97)	102 (88.69)	276,590	2826	117,914 (87.65)	154 (95.06)	241,166	3327
ChAdOx1 nCoV-19	2594 (1.86)	2 (1.74)	276,590	2826	0	N/A	241,166	3327
BNT162b2	9836 (7.03)	6 (5.22)	276,590	2826	9584 (7.12)	4 (2.47)	241,166	3327
Gam-COVID-Vac	7192 (5.14)	5 (4.35)	276,590	2826	7037 (5.23)	4 (2.47)	241,166	3327
All vaccines	139,858 (100)	115 (100)	276,590	2826	134,535 (100)	162 (100)	241,166	3327

N = number; N/A = not applicable
